# Exploring paraptosis as a therapeutic approach in cancer treatment

**DOI:** 10.1186/s12929-024-01089-4

**Published:** 2024-11-04

**Authors:** Ling-Chu Chang, Shih-Kai Chiang, Shuen-Ei Chen, Mien-Chie Hung

**Affiliations:** 1 Center for Molecular Medicine, China Medical University Hospital, Taichung, 406040 Taiwan; 2 Research Center for Cancer Biology, China Medical University, Taichung, 406040 Taiwan; 3 Cancer Biology and Precision Therapeutics Center, China Medical University, Taichung, 40402 Taiwan; 4 Department of Animal Science, National Chung Hsing University, Taichung, 40227 Taiwan; 5 The iEGG and Animal Biotechnology Center, National Chung Hsing University, Taichung, 40227 Taiwan; 6 Innovation and Development Center of Sustainable Agriculture (IDCSA), National Chung Hsing University, Taichung, 40227 Taiwan; 7 i-Center for Advanced Science and Technology (iCAST), National Chung Hsing University, Taichung, 40227 Taiwan; 8 Graduate Institute of Biomedical Sciences, China Medical University, Taichung, 406040 Taiwan

**Keywords:** Paraptosis, Cancer cells, Proteasome inhibition, Endoplasmic reticulum stress, Mitochondria, Ion homeostasis, Reactive oxygen species

## Abstract

A variety of cell death pathways play critical roles in the onset and progression of multiple diseases. Paraptosis, a unique form of programmed cell death, has gained significant attention in recent years. Unlike apoptosis and necrosis, paraptosis is characterized by cytoplasmic vacuolization, swelling of the endoplasmic reticulum and mitochondria, and the absence of caspase activation. Numerous natural products, synthetic compounds, and newly launched nanomedicines have been demonstrated to prime cell death through the paraptotic program and may offer novel therapeutic strategies for cancer treatment. This review summarizes recent findings, delineates the intricate network of signaling pathways underlying paraptosis, and discusses the potential therapeutic implications of targeting paraptosis in cancer treatment. The aim of this review is to expand our understanding of this unique cell death process and explore the potential therapeutic implications of targeting paraptosis in cancer treatment.

## Introduction

Programmed cell death plays an important role in various processes, such as tissue development, differentiation, and homeostasis. Dysregulation of programmed cell death leads to pathogenesis, including cancer, neurodegenerative diseases, autoimmune disorders, and cardiovascular diseases. With advancements in molecular technologies, several types of cell death have been identified and characterized. Paraptosis, a novel type of programmed cell death, was first identified by Sperandio et al. in 2000 [1]. They reported that human embryonic kidney 293 T cells and mouse embryonic fibroblasts overexpressing the intracellular domain of the β subunit of insulin-like growth factor 1 receptor (IGF1R) underwent cell death characterized by a distinctive vacuole-filled cytoplasm morphology, unlike other known forms of cell death at that time. They termed this form of cell death “paraptosis,” using the Greek preposition “para” to distinguish it from apoptosis because paraptosis does not involve caspase activation, apoptotic body formation, DNA fragmentation, or chromatin condensation. Over the past two decades, some of the underlying mechanisms of paraptosis have been defined, revealing its association with cell fate and various diseases, such as cancer [2–4] and viral infection [5]. Furthermore, some small molecules that induce paraptosis have shown potential as anticancer agents by inhibiting tumor growth and overcoming therapeutic resistance [2–4, 6, 7]. This makes paraptosis an attractive alternative for cancer treatment. In this review, we summarize the discoveries of paraptosis, with an emphasis on its role in cancer biology, including its specific features, regulatory mechanisms, and potential applications in cancer treatment, in the hope of providing insights into the development of effective clinical anticancer strategies.

## Characteristics of paraptosis

### Morphological features of paraptosis

**Fig. 1:**
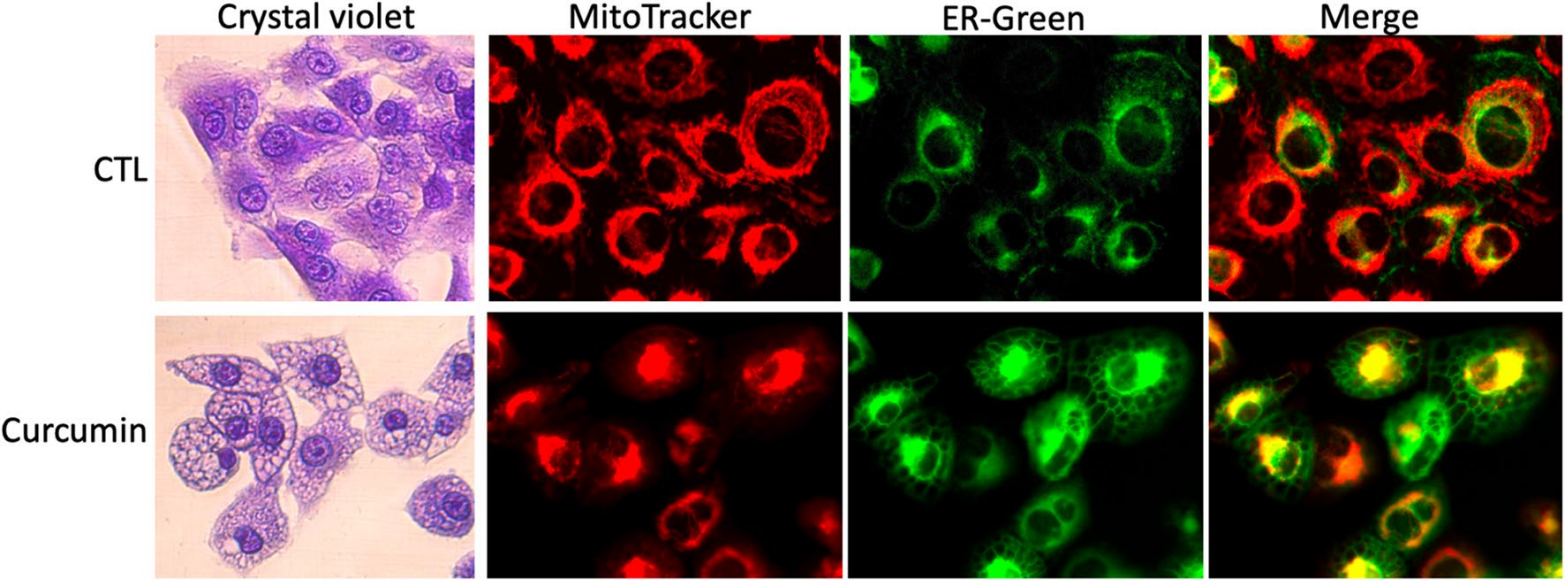
Curcumin induces distinct paraptotic morphological alterations in MDA-MB-435 melanoma cells. MDA-MB-435 cells were treated with 30 μM curcumin (Cat#81025, Cayman Chemicals) for 24 h. Then, cytoplasmic vacuolization was visualized using crystal violet staining. Vacuolization was also visualized by fluorescent microscopy using co-staining of mitochondria with MitoTracker® Red CMXRox (Cat # 9082, Cell Signaling Technology) and the endoplasmic reticulum with ER-tracker™ Green (Cat#E34251, ThermoFisher Scientific) (Chang, unpublished data)

Cytoplasmic vacuolization is the hallmark of paraptosis. Cytoplasmic vacuoles in paraptosis originate from swelling of the endoplasmic reticulum (ER) and mitochondria. Paraptotic cells maintain plasma membrane integrity but exhibit enlarged cell sizes because the vacuoles fill the cytosol. As shown in Fig. 1, curcumin, a known paraptotic agent [8], induces dilation of both the ER and mitochondria. The ER and mitochondria are the primary intracellular storage sites for calcium ions (Ca^2+^). It has been proposed that ER Ca^2+^ influx into mitochondria triggers water influx into the ER [9], leading to ER dilation to accommodate the increased volume, followed by mitochondrial dilation. Eventually, paraptotic cells detach, round up, shrink, and rupture the outer membrane [2, 8, 10, 11]. Cytoplasmic vacuolization is also observed in two other types of cell death, autophagy and methuosis [11–15]. Macroautophagic vacuoles originate from elongating isolated membranes. In macroautophagy, cytosolic membranes, or phagophores, elongate and seal to form a double-membraned autophagosome, subsequently encapsulating cytosolic components either selectively or nonselectively. The autophagosome subsequently fuses with lysosomes, degrading their contents [16–18]. The role of macroautophagy in response to ER stress is complex and context dependent, with studies reporting both cytoprotective and cell death-inducing effects [17, 18]. Methuosis, another form of vacuolar cell death, originates from hyperactive micropinocytosis. The accumulation of macropinosomes and endosomes results in a characteristic vacuolated morphology, ultimately culminating in cell death [11, 19–21]. The similarity and difference in paraptosis, macroautophagic death, and methuosis are listed Table 1.

**Table 1:** The comparison of paraptosis, autophagy, and methuosis

Features	Paraptosis	Macroautophagic death	Methuosis
First appearance	Sperandio et al., 2000	Clark et al., 1957 Novikoff et al., 1959	Overmeyer et al., 2008 Bhanot et al., 2010
Nomenclature	*para*, relate to; *ptosis*: falling off	*phagy*, eat; *auto*: self	*methuo*, to drink to intoxication
Morphology under microscope	Cytoplasmic vacuolation	Cytoplasmic vacuolation	Cytoplasmic vacuolation
Cell swelling	Yes	Yes	Yes
Rupture of plasma membrane	Yes	Yes	Yes
Vacuole membrane construction	Swelled endoplasmic reticulum and mitochondria	Autophagosomes	Macropinosomes, endosomes
Vacuole marker and staining	Endoplasmic reticulum dye: ER-tracker, Mitochondrial dye: MitoTracker	LC3 staining	Rab5, Rab 7, EEA staining
Vacuole contents	Primarily water and electrolytes, misfolded/unfolded proteins, damaged organelles Dilated ER and mitochondria can be engulfed by autophagosomes, which then fuse with vacuoles, contributing to their contents	Degraded cytoplasmic materials: proteins, organelles, lipids Misfolded protein-formed aggregates Intracellular pathogens	Extracellular fluid with its components, such as water, ions, and nutrients Limited cytoplasmic contents
Vacuole movement	No	Yes	Yes
Caspase-dependent	No	No	No
Chromatin condensation	No	No	No
DNA fragmentation	Varies	No	Yes (late stage)
Activation signaling or biomarkers	–	LC3-II lipidation, p62 degradation	Ras, Rac1
Mitochondrial membrane permeability and potential	Yes	Yes	Varies
Accumulation of autophagosome	Yes	Yes	Yes

Reference: [1–4, 6, 7, 10–21]

### Biochemical features

#### Disruption of ion homeostasis

Dilation of the ER and mitochondria results from an imbalance of calcium within these organelles [2, 10, 11]. Calcium is critical for the proper folding of proteins within the ER. Depletion of ER calcium leads to ER stress, activation of the unfolded protein response (UPR), and the accumulation of misfolded or unfolded proteins [22]. This accumulation of misfolded proteins in the ER increases osmotic pressure, activating inositol 1,4,5-triphosphate receptors (IP3Rs) to promote Ca^2+^ release from the ER into the cytosol and mitochondria [9, 23, 24]. The resulting osmotic pressure drives water influx into the ER, causing ER dilation. Similarly, an ionic imbalance in mitochondria increases osmotic pressure and water influx, leading to mitochondrial swelling [25, 26].

#### Proteasomal inhibition, ER stress,  UPR

Most paraptotic agents possess proteasomal inhibition activity, which prevents the degradation of ubiquitin-tagged proteins and results in the accumulation of these proteins [4, 8, 9, 11]. Impaired proteasome function leads to the accumulation of misfolded or damaged proteins in the ER, triggering ER stress and the subsequent UPR. As a result of ER stress, relevant proteins are often upregulated [8, 9, 27–36]. These proteins not only accumulate in the ER but also translocate to the nucleus, where they participate in transcription activation and translation [29, 30, 36]. Therefore, inhibitors of transcription and translation can mitigate cell vacuolization [8, 9, 26, 35–38].

#### Reactive oxygen species (ROS) production

ROS overproduction is often observed in paraptotic cells [30, 36, 39–42]. ROS production could be caused by proteasome inhibition [8, 9, 37, 43], mitochondrial dysfunction [37, 42, 43], and redox system imbalance [30, 39, 40]. Notably, ROS themselves act as signaling molecules that mediate paraptosis [30, 39–42]. A more detailed mechanism of ROS crosstalk with paraptosis is described in the following section.

## Paraptosis-inducing molecules and associated signaling pathways

**Fig. 2:**
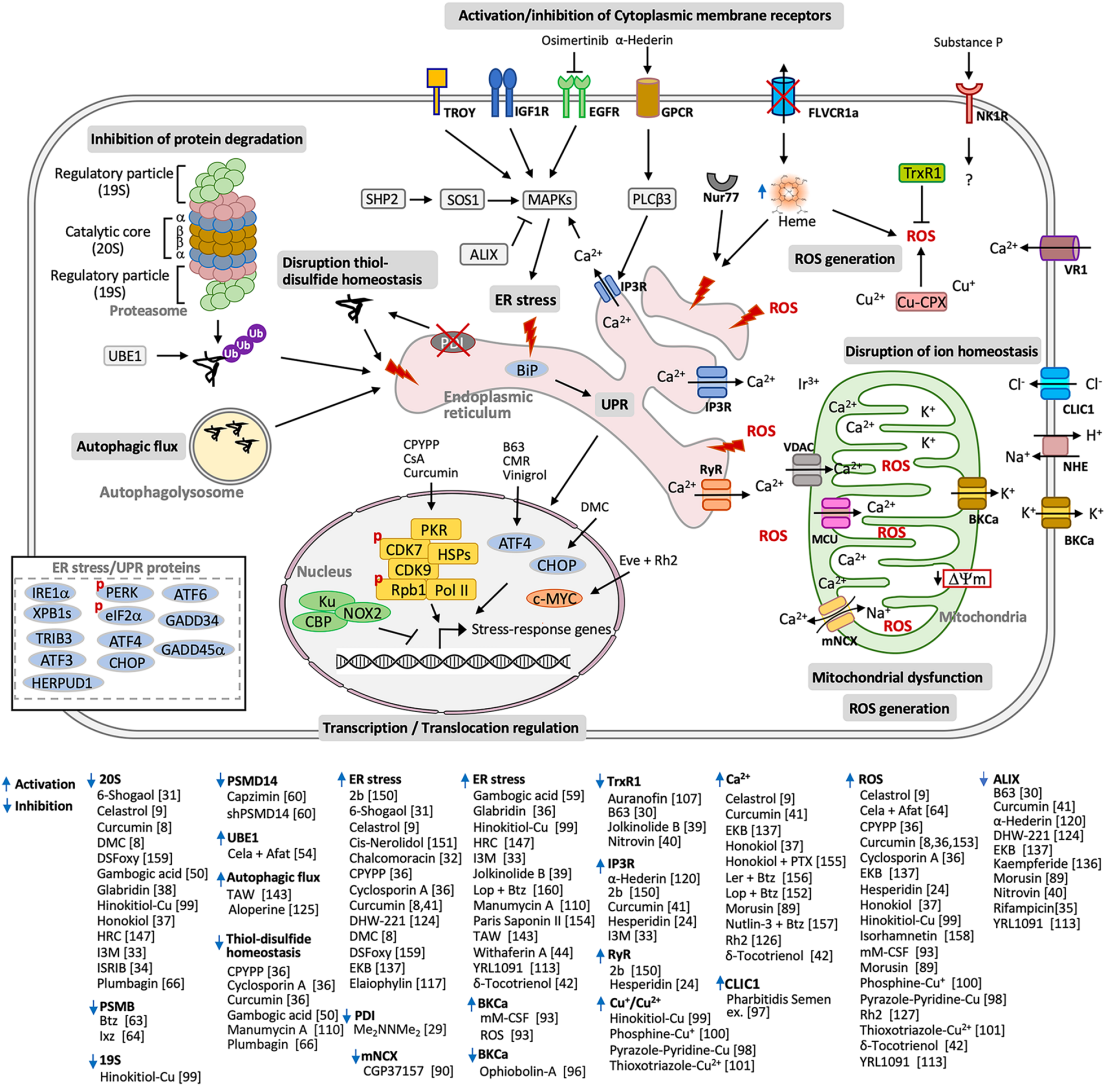
This figure illustrates the complex network of signaling pathways involved in paraptosis, highlighting the key small molecules and proteins that regulate them. Pathways leading to paraptosis include inhibition of proteasomal degradation, disruption of thiol-disulfide homeostasis, and induction of endoplasmic reticulum (ER) stress and the unfolded protein response (UPR). Additionally, paraptosis can be triggered by disruption of cellular calcium and/or other ion homeostasis, mitochondrial dysfunction and overproduction of reactive oxygen species (ROS), interference with autophagic flux, and activation of transcription and translation. Furthermore, the activation or inhibition of receptors, such as TROY, IGF1R, EGFR, and GPCRs, and their associated signaling pathways can modulate paraptosis. Proteasome inhibition leads to the accumulation of ubiquitinated proteins, contributing to ER stress and consequently activating the UPR. ER stress and UPR activation increase transcription and translation activities. This severe ER stress disrupts calcium homeostasis, causing Ca^2+^ efflux from the ER into the mitochondria. This efflux leads to mitochondrial dysfunction and an imbalance of intracellular redox homeostasis, resulting in ROS overproduction. Elevated ROS levels and imbalanced ER Ca^2+^ levels further exacerbate ER stress. Some ER and UPR proteins also serve as transcription factors that promote the expression of other UPR proteins. The lower left panel shows the known upregulated ER stress/UPR proteins during paraptosis. The table below presents the compounds and treatments that induce paraptosis and their respective signaling pathways. Upward arrows indicate activation, while downward arrows indicate inhibition. References to the effects of small molecules are indicated within square brackets. Abbreviations for some small molecules are provided: 2b, isoxazole derivative of usnic acid; 4a, N-(2-hydroxyethyl)formamide compound; B63, curcuminoid B63; Btz, bortezomib; Cela, celastrol; DMC, dimethoxycurcumin; DSFoxy, disulfiram oxy-derivatives; EKB, epimedokoreanin B; Eve, everolimus; HRC, 2’-hydroxy-retrochalcone; I3M, indirubin-3’-monoxime; Ixz, ixazomib; Ler, lercanidipine; Lop, loperamide; Pharbitidis semen ex., Pharbitidis semen extract; Rh2, ginsenoside Rh2; ROS, reactive oxygen species; TAW, 8-p-hydroxybenzoyl tovarol

To date, specific pathways or unique molecular biomarkers for paraptosis remain elusive. Instead, multiple interconnected pathways have been implicated in initiating paraptosis. This section summarizes paraptosis-inducing compounds and treatments, highlighting their downstream effectors and associated signaling pathways (Fig. 2).

### Proteasomal inhibition

In cells, protein degradation is carried out via two systems: the ubiquitin‒proteasome system (UPS) and the autophagy‒lysosome system. Autophagy, a self-eating process, degrades cellular components such as organelles or aggregated proteins by engulfing substrates in a double-membrane compartment called the autophagosome, which then fuses with lysosomes [16, 17, 45]. Unlike the autophagy‒lysosome system, which degrades a wide range of cellular components, the proteasome primarily targets signaling proteins and misfolded or unfolded proteins. The proteasome is distributed in both the cytosol and the nucleus and is essential for maintaining cellular protein homeostasis [46, 47]. Proteasome activity is subject to extensive regulation, encompassing processes ranging from cell proliferation and growth to stress responses. Therefore, the proteasome not only maintains cellular amino acid and protein homeostasis but also controls a variety of pivotal cellular activities, including the cell cycle, transcription, DNA replication, signal transduction, and stress responses [48, 49].

Deficiencies in proteasomal activity are key factors contributing to paraptosis induction [4, 9, 11, 31, 33, 37, 38, 50]. Proteasome inhibition impedes protein degradation and recycling, leading to the accumulation of misfolded proteins in the ER and subsequent UPR activation [51, 52]. Severe protein misfolding results in ER-associated degradation (ERAD), where misfolded proteins are translocated to the cytosol for proteasomal degradation [53]. The accumulation of ubiquitinated proteins triggers various cellular stress responses, including ER overload, oxidative stress, and impaired protein function, ultimately contributing to paraptosis and apoptosis [4, 11, 54].

The proteasome of eukaryotic cells is composed of two subcomplexes: the 19S regulatory particle and the 20S catalytic core particle. The 19S regulatory particle functions primarily to recognize polyubiquitinated substrates, deubiquitinate them, and transport the substrates into the core particle, which houses the proteolytic site of the proteasome. Proteasome inhibitors reversibly or irreversibly bind to the proteasome's catalytic sites, inhibiting its chymotrypsin-, trypsin-, and caspase-like activities [46, 47, 52, 55]. Bortezomib targets the β5 and β1 subunits of the 20S proteasome core, inhibiting both chymotrypsin- and caspase-like activity [56], while ixazomib specifically inhibits the chymotrypsin-like activity of the β5 subunit [57]. Bortezomib induces ER stress by upregulating chaperones (BiP/GRP78, gp96/GRP94) and activating UPR signaling (PERK, ATF4, CHOP) [58]. PSMD14, a deubiquitinating subunit of the 19S proteasome, is crucial for proteasome function [59]. Its depletion, induced by capzimin or shRNA, triggers paraptosis with concomitant ER-to-cytosol Ca^2+^ flux [60]. The inhibition of 20S proteasome activity by several small molecules plays a role in the mechanism of paraptosis induction, including honokiol [37], 6-shogaol [31], gambogic acid [50], ezetimibe [61], plumbagin [62], dimethoxycurcumin (DMC), and curcumin [8]. UBE1, the ubiquitin-activating enzyme that initiates protein ubiquitination [63], also influences this process. UBE1 knockdown attenuates celastrol- and afatinib-induced protein ubiquitination, cytoplasmic vacuolization, and cell death [64]. Proteolytic inhibition of proteasome can potentiate anticancer effects by priming cell paraptosis. For example, bortezomib enhances the action of ISRIB, a small molecule that restores eIF2B-mediated translation, by shifting cells from apoptosis to paraptosis [65].

Mitochondrial dysfunction is another critical effect observed when proteasome function is disrupted. Blockade of proteasome activity can trigger a compensatory stress response to restore mitochondrial homeostasis. This response involves the activation of mitochondrial quality control mechanisms, such as the mitochondrial unfolded protein response (UPR^mt^), mitophagy, and the upregulation of chaperone proteins [66]. Following the inhibition of proteasome activity, mitochondrial impairment increases electron leakage and ROS generation, leading to the exacerbation of cytosolic oxidative stress and cell death [43]. Dysregulation of mitochondrial dynamics, including fusion, fission, and motility, by inactivating proteasomes can lead to alterations in mitochondrial morphology and disruptions within cells. Disruption of the mitochondrial membrane potential due to proteasome inactivation can impair ATP production and calcium homeostasis, leading to the induction of death signaling [67].

Proteasome function is also related to cell cycle progression. Cell cycle arrest occurs due to the accumulation of cell cycle regulatory proteins such as cyclins, cyclin-dependent kinase (CDK) inhibitors, and cell cycle inhibitors, which are normally targeted for degradation by proteasomes [68]. The proteasome inhibitor bortezomib has been shown to increase the levels of p21, p27, and cyclin B1, leading to G_2_/M cell cycle arrest and the activation of apoptotic signaling in glioblastoma cells and T lymphoblastic leukemia cells [69, 70]. However, whether proteasomal inhibition-induced cell cycle arrest is involved in paraptosis signaling remains to be explored. Furthermore, an increased level of nuclear proteasomes was observed during paraptosis, suggesting proteolytic stress within the nucleus [36]. However, its role in the stress response, whether it is recruited into the nucleus to ameliorate proteotoxicity locally or to target ubiquitinated proteins for degradation within the nucleus, remains unknown. Nevertheless, given the role of proteasomes in maintaining cellular proteostasis, UPS can influence cell fate toward survival or death by regulating signaling effectors in the cytoplasm and nucleus [54, 71, 72].

### ER/nuclear stress and the UPR

The ER is the primary organelle responsible for protein synthesis, folding, and sorting. ER-localized proteins assist in this synthesis process. The accumulation of misfolded or unfolded proteins can induce ER stress, followed by the UPR, which functions to restore ER homeostasis by decreasing the load of misfolded proteins and promoting cell survival through a set of transcriptional and translational events. However, if the level of accumulated proteins far exceeds the cell’s capacity to restore homeostasis, ER stress can become prolonged and severe, leading to the induction of cell death [73, 74]. The downstream effects of ER stress also include ER-associated degradation, autophagy, oxidative stress, mitochondrial dysfunction, and metabolite dysregulation [75].

The UPR comprises three signaling pathways activated by the accumulation of misfolded proteins in the ER, with the chaperone protein binding immunoglobulin protein (BiP) serving as an initial sensor [22]. BiP initiates the UPR by dissociating from three luminally located proteins: protein kinase RNA-like ER kinase (PERK), inositol-requiring enzyme 1α (IRE1α), and activating transcription factor 6 (ATF6) [22]. PERK and IRE1α are then activated via multimerization and trans-autophosphorylation, while ATF6 translocates to the Golgi apparatus, where it is proteolytically cleaved into the soluble and active form, the ATF6 transcription factor [76]. As sensors, PERK, IRE1α, and ATF6 further engage in three distinct UPR signaling pathways. Active PERK phosphorylates the α subunit of the eukaryotic initiation factor eIF2 to selectively activate the translation of certain proteins, including ATF4, DNA damage-inducible protein 34 (GADD34), and C/EBP homologous protein (CHOP) [22]. ATF4 regulates the expression of genes involved in amino acid biosynthesis, protein folding, antioxidant responses, and redox balance. Under persistent stress, ATF4 promotes autophagy and transcriptionally activates CHOP to induce cell death. GADD34 mediates the dephosphorylation of eIF2 to restore protein synthesis. IRE1α functions as an endoribonuclease to generate the active spliced isoform of the X-box binding protein transcription factor (XBP1) [77]. XBP1 modulates the expression of proteins and chaperones involved in ER membrane biosynthesis, lipid biosynthesis, protein folding, and the ER-associated degradation process [78]. IRE1α also activates c-Jun N-terminal kinase (JNK) to promote autophagy and apoptosis [79]. ATF6 triggers transcriptional programs to restore ER homeostasis, including upregulating BiP and chaperone expression, enhancing ERAD, and promoting the *N*-glycosylation of proteins. ATF6 also induces CHOP expression, contributing to UPR-related cell death [80].

In the paraptosis process, BiP, ER stress sensor proteins (IRE1α, PERK, ATF6), and response marker proteins (CHOP, ATF3, ATF4, GADD34, GADD45α, HERPUD1, TRIB3) are significantly upregulated [8, 9, 27–33, 35, 36]. These findings suggest a complex interplay between ER stress-induced rescue and cell death programs involving multiple protein responses. ER stress response proteins, such as ATF3, ATF4, and CHOP, also function as transcription factors or cofactors to amplify UPR signaling [75]. BiP knockdown attenuated chalcomoracin-induced vacuolization and cell proliferation [32]. ATF4 knockdown decreased the paraptosis induced by the anticancer agent curcuminoid B63 in gastric cancer cells [30]. The knockout of ATF4 or CHOP delayed paraptosis induced by the diterpenoid vinigrol in MCF-7 breast cancer cells [81]. Moreover, knockdown of CHOP markedly attenuated dimethoxycurcumin-induced ER dilation and cell death in breast cancer cells [8]. Pharmacological eIF2α inhibitors, salubrinal and ISIRB, targeting the PERK-eIF2α axis, impaired paraptosis induced by the EGFR inhibitor osimertinib [34]. Interestingly, these ER sensors and effectors, including IRE1α, PERK, BiP, HERPUD1, GADD34, and TRIB3, also accumulate in the nucleus [36], suggesting the occurrence of nuclear stress. Whether their nuclear accumulation contributes to relieving proteolytic stress or targeting transcriptional events needs further investigation.

### The perturbation of ion homeostasis and osmotic pressure

ER and mitochondrial dilation, hallmarks of paraptosis, are linked to the dysregulation of ion homeostasis involving Ca^2+^, potassium (K^+^), chloride (Cl^−^), and copper (Cu^+^, Cu^2+^) ions [9, 10, 29, 82, 83]. The ER and mitochondria play pivotal roles in intracellular Ca^2+^ homeostasis, serving as storage depots and responding to cytosolic Ca^2+^ signals [84]. Optimal protein folding machinery function is dependent on Ca^2+^, with chaperones and folding enzymes such as BiP, protein disulfide isomerase, GRP94, and HSPs regulated by Ca^2+^ binding [23, 84–86] ER membrane-embedded IP3Rs and RyR channels control Ca^2+^ release [87, 88], and their activation can disrupt ER Ca^2+^ levels, contributing to ER stress, protein misfolding, and subsequent dilation [23]. Misfolded protein accumulation within the ER lumen generates osmotic pressure, driving water influx and ER distension to dilute the protein concentration [22, 51].

When the ER is constitutively swollen under stress, it promotes the release of calcium from the ER to the mitochondria through the ER‒mitochondrion axis via an intracellular Ca^2+^ flux mechanism [10]. Voltage-dependent anion channels (VDACs) mediate δ-tocotrienol and morusin-induced mitochondrial Ca^2+^ load and ROS generation, leading to subsequent paraptotic induction [42, 89]. Indirubin-3’-monoxime (I3M), a synthetic indirubin derivative, inhibits proteasomal activity, causes ER stress by promoting Ca^2+^ influx into mitochondria through mitochondrial Ca^2+^ uniporters (MCUs), and thus induces paraptosis in MDA-MB-231 breast cancer cells [33]. The cyclometalated iridium complex and the mitochondrial Na^+^/Ca^2+^ exchanger (mNCX) inhibitor CGP37157 induce paraptosis in Jurkat cells by promoting mitochondrial–ER membrane fusion, leading to subsequent ER-to-mitochondrial Ca^2+^ influx and decreased mitochondrial membrane potential [90]. Mitochondrial Ca^2+^ overload elicits ROS production, which in turn promotes the opening of the mitochondrial permeability transition pore (mPTP) [91]. Owing to an influx of water, ions, and other solutes into the matrix, mitochondrial dilation can equilibrate the pressure inside the mitochondrial inner membrane and diminish the mitochondrial membrane potential, subsequently inhibiting the coupling of oxidative phosphorylation and ATP production [25]. Due to the loss of the mitochondrial membrane potential, mitochondria fail to retain Ca^2+^, leading to increased cytosolic Ca^2+^ levels. ATP depletion hinders ER pump Ca^2+^ from the cytosol using SERCA (sarcoplasmic/endoplasmic reticulum calcium ATPase), exacerbating misfolded protein accumulation and ER dilation. The overexpression of vanilloid receptor subtype 1 (VR1), which is located on the cytoplasmic membrane, was shown to promote Ca^2+^ uptake and induce sustained increases in [Ca^2+^], leading to mitochondrial dysfunction and paraptosis [92].

The killing of human glioma cells expressing membrane macrophage colony-stimulating factor (mM-CSF) by monocytes/macrophages was shown to occur via the activation of large conductance Ca^2+^-activated K^+^ channels (BKCa) on the cell membrane, ER, and mitochondria [93]. The opening of BKCa channels expels K^+^ stores, while sodium (Na^+^) and water enter the cells, causing the ER and mitochondria to swell and form vacuoles [93]. Furthermore, exposure to ROS can also lead to K^+^ expulsion from cells, prompting the opening of BKCa channels in the plasma membrane, ER, and mitochondrial membrane. Consequently, Na^+^ and water flux into the cells to maintain electrical neutrality, further exacerbating cellular swelling. This swelling eventually culminates in cell lysis, releasing heat shock proteins (HSPs) and high mobility group box 1 (HMGB1/amphoterin) proteins [94]. HMGB1, a nuclear protein, has diverse functions depending on its location, binding partners, and oxidation state [95]. When located in the periphery, HMGB1 serves as a significant indicator of paraptosis induction in T9 glioma cells of mice. In this context, HMGB1 functions as a danger signal to potentially elicit immune responses against tumors [94]. Interestingly, inhibiting BKCa channels can also induce cell paraptosis. Ophiobolin A, an inhibitor of calmodulin-activated cyclic nucleotide phosphodiesterase, inhibits BKCa activity and leads to the accumulation of K^+^ in the ER and mitochondria. This disrupts osmotic pressure and causes cytoplasmic vacuolization in glioblastoma multiforme cells. BKCa inhibition also alters the dynamic organization of the F-actin cytoskeleton [96].

Elevated intracellular Cl^−^ concentrations can activate chloride intracellular channel 1 (CLIC1), leading to paraptosis. A purified resin glycoside fraction from *Pharbitidis semen* induces paraptosis in human colon cancer cells through the activation of CLIC1 [97]. Copper overload-induced cytotoxicity also includes paraptotic programs. In HT-1080 cells, pyrazole‒pyridine copper complexes increase ER stress and inhibit caspase 3. ER stress, potentially induced by UPS inhibition [98], may contribute to paraptosis. Elevated copper levels, resulting from the hinokitiol-copper complex, trigger paraptosis by specifically targeting the 19S proteasomal deubiquitinase, leading to impaired 20S proteasome activity in lung cancer A549 and leukemia K562 cells. Additionally, hinokitiol-copper-induced paraptosis depends on ATF4-associated ER stress [99]. Other copper complexes, such as phosphine copper (I) and thioxotriazole copper (II) complexes, also inhibit 20S proteasome activity and induce ER stress to achieve anticancer effects [100, 101]. In addition, copper accumulation enhances paraptosis effects through the provocation of ROS or by activating paraptosis-related signaling pathways, such as the MAPK and phosphoinositide 3-kinase (PI3K)–AKT pathways [99, 102]. The combination of copper and 8-hydroxyquinoline induces paraptosis in HeLa cells via ROS generation and activation of the UPR [103]. After endocytosis, disulfiram-loaded Ca^2+^/Cu^+^ dual-ion nanotraps exhibit similar enhancements in cell paraptosis [104]. Additionally, MnO_2_ encapsulated within nanoparticles can serve as an oxygen modulator to amplify the effect of paraptosis agents [105]. The increase in constitutive ferritinophagy by NCOA4 results in an increase in the level of intracellular ferrous iron (Fe^2+^), which in turn elevates ROS production and activates the MAPK pathway to prime cell paraptosis [106].

### ROS generation and redox regulation

ROS provocation is a hallmark of paraptosis development and is apparently a secondary effect due to deficient proteasome activity, ER stress, the UPR, and elevated mitochondrial Ca^2+^ levels [20, 54]. ROS is essential for many small molecules that induce paraptosis, such as curcumin, dimethoxycurcumin [8], honokiol [37], curcuminoid B63 [30], δ-tocotrienol [42], jolkinolide B [39], auranofin [107], nitrovin [40], and CPYPP [36]. ROS generation in paraptosis primarily stems from mitochondrial dysfunction and disruption of antioxidant systems, including thioredoxin and glutathione (GSH) systems [30, 39, 40]. Mitochondria, the primary sites of energy production and major sources of ROS generation, are critically damaged during paraptosis. This damage is fatal due to their pivotal role in cellular energy metabolism. Mitochondrial dysfunction, manifested as membrane potential dissipation and the uncoupling of oxidative phosphorylation, promotes ROS generation [108]. δ-Tocotrienol downregulates OXPHOS complex I, inhibiting oxygen consumption and mitochondrial membrane potential, thereby hindering ATP synthesis and resulting in ROS overproduction [42]. Withaferin A (WA) treatment induced hyperpolarization of mitochondrial membrane potential and cytoplasmic vacuolation in breast cancer MCF-7 and MDA-MB-231 cells [44]. Proteasome inhibition enhances electron leakage from the electron transport chain, increasing ROS production [43]. Mitochondrial ROS increase can increase mitochondrial Ca^2+^ levels. Hesperidin increased mitochondrial Ca^2+^ levels, mediated by ROS increase, leading to ERK activation and subsequent vacuolization in hepatoblatoma HepG2 cells [24].

Thioredoxin reductase (TrxR1) is a key regulator of redox balance in mammalian cells, preventing oxidative stress [109]. The paraptosis induced by curcuminoid B63 was mediated by ROS-mediated ER stress and MAPK activation through targeting TrxR1 in gastric cells [30]. In vivo studies have shown that B63 treatment reduced the growth of gastric cancer tumors, associated with an increase in ROS to induce paraptosis [30]. Jolkinolide B, nitrovin, and auranofin promote ROS generation by targeting TrxR1 [39, 40, 107]. GSH is the most abundant antioxidant in cells, and its depletion also leads to an increase in ROS [39, 40]. In addition to TrxR1 inhibition, Jolkinolide B also targets the GSH system to induce ROS-mediated paraptosis and apoptosis in bladder cancer cells [39]. Auranofin acts by dually inhibiting TrxR1 and the proteasome, upregulating the ATF4–CHAC1 axis to degrade GSH, consequently aggravating proteotoxic stress and promoting paraptosis [106]. ROS overproduction activates MAPK and mTOR pathways [39, 40]. In CPYPP-induced paraptosis, ROS overproduction enhances CDK7 and CDK9 activity, consequently upregulating heat shock protein expression and protein kinase R [36].

*N*-acetylcysteine, a thiol-containing compound, effectively rescues cell paraptosis under the induction of plumbagin [62], manumycin A [110], gambogic acid [50], and CPYPP [36], suggesting the involvement of thiol-disulfide homeostasis in the development of paraptosis. Thiol-disulfide homeostasis refers to the dynamic balance between thiol (-SH) groups and disulfide (S‒S) bonds within a cell, which functions critically in maintaining the cellular redox status and proper protein function. This system is regulated mainly by GSH, thioredoxin, and protein disulfide isomerase (PDI) [111]. The thiosemicarbazone Me_2_NNMe_2_ induces cell paraptosis by disrupting ER thiol redox homeostasis through the inhibition of PDI activity [29]. *N*-acetylcysteine can antagonize the blockade of proteasome activity [112], suggesting that thiol-disulfide homeostasis governs proteasome function for proteolysis. Therefore, impeded thiol–disulfide homeostasis apparently acts upstream, preceding the occurrence of proteolytic stress and proteotoxicity (Table 2).

**Table 2:** The effects of reactive oxygen species (ROS) in paraptosis

Small molecules or treatments	Cancer types	Causes of ROS increase	Affected signaling pathways	References
Auranofin	Breast cancer cells (MDA-MB-435S)^#^	Inhibit TrxR1 enzyme activity, proteasome activity Decrease GSH levels	Induce ER stress (upregulate p-eIF2α, CHOP) Activate ATF4/CHAC1 axis leads to GSH degradation	[107]
Curcuminoid B63	Gastric cancer cells (SGC-7901, BGC-823, SNU-216)	Inhibit TrxR1 activity	Induce ER stress (upregulate p-eIF2α, CHOP, ATF4) Activate MAPK pathway (p38, ERK, JNK)	[30]
CPYPP Curcumin Cyclosporin	Melanoma (MDA-MB-435), breast cancer cells (MDA-MB-231)	Impair thiol homeostasis	Induce ER stress (upregulate CHOP, ATF3, ATF4, GADD34, GADD45α, PERK, IRE1α, HERPUD1, BiP) Increase CDK7 and CDK9 activity Upregulate protein kinase K, heat shock proteins	[36]
δ-Tocotrienol (δ-TT)	Melanoma cells (A375, BLM)	Induce Ca^2+^ overload Downregulate OXPHOS complex I, decrease oxygen consumption and mitochondrial membrane potential	Activate MAPK (p38, ERK, JNK) Inhibit ATP synthesis	[42]
Jolkinolide B	Bladder cancer cells (T24, UM-UC-3)	Inhibit TrxR1 enzyme activity without change protein, depletion of glutathione	Induce ER stress (upregulate ATF4, CHOP) Activate MAPK (p38, ERK, JNK)	[39]
Nitrovin	Glioblastoma multiforme (U251, U87)	Inhibit thioredoxin reductase (TrxR1) activity without changing TrxR1 protein	Induce ER stress (upregulate p-eIF2α, CHOP, ATF4, BiP) Activate MAPK (p38, ERK, JNK)	[40]
Me_2_NNMe2	Colorectal cancer (SW480, HCT-116)	Inhibit disulfide isomerase (PDI) activity and disturb endoplasmic reticulum thiol redox homeostasis	Activate MEK–ERK pathway Induce ER stress (upregulate PERK, p-eIF2α, ero1L-α, CHOP)	[29]
Withaferin A	Breast cancer lines: MCF-7, MDA-MB-231	Promote the hyperpolarization of mitochondrial membrane potential	Induce ER stress (upregulate CHOP, BiP)	[44]
Honokiol	Acute promyelocytic leukemia (NB4 cells)	Promote the depolarization of mitochondrial membrane potential	Induce ER stress (upregulate Bip, CHOP) Activate mTOR, MAPK (p38, ERK, JNK)	[37]
YRL1091, pyrazolo[3,4-*h*]quinoline derivative	Breast cancer cells (MDA-MB-231, MCF-7)	Increase ROS	Induce ER stress (upregulate ATF4, CHOP, BiP) Activate MAPK (ERK, JNK)	[113]

^#^MDA-MB-435S was previously classified as breast ductal carcinoma. However, ATCC has reclassified it as melanoma. https://www.atcc.org/products/htb-129

During the progression of paraptosis, elevated mitochondrial ROS production contributes to the impairment of proteasome activity. For example, in celastrol-mediated paraptosis, ROS production due to mitochondrial dysfunction caused by Ca^2+^ overload can impair proteasome activation [9]. δ-Tocotrienol-induced mitochondrial Ca^2+^ overload and ROS generation, followed by MAPK activation, contribute to paraptosis in melanoma cells [42]. This impairment could stabilize IP3R and the mitochondrial calcium uniporter (MCU), thereby reinforcing Ca^2+^-mediated effects, leading to ER stress, vacuolization, and eventually cell death [9]. Collectively, these results suggest that deficient proteasome activity and ROS provocation work together to create a positive feedback loop, amplifying oxidative signaling and leading to cell paraptosis.

### IGF1R and other cytoplasmic membrane receptors in paraptotic induction

IGF1R activation was initially implicated in the induction of paraptosis via the MAPK/ERK and JNK pathways [1]. Alg-2-interacting protein (ALIX/AIP) acts as an endogenous paraptotic inhibitor by modulating MAPK activation [115], while RKIP, a Raf kinase inhibitor, attenuates paraptosis by suppressing JNK signaling [116]. Additionally, MAPKs mediate SHP2-induced paraptosis [117]. Pharmacological inhibition of TrxR1 and GSH depletion by jolkinolide B can augment ROS production, activating the MAPK pathway and priming cells for paraptosis in bladder cancer [39]. In contrast, iodine-125 seed radiation induces paraptosis via the PI3K/AKT pathway in colorectal cancer cells (HCT116), preceding MAPK activation [114].

Other receptors, such as TROY/TAJ/TNF receptor superfamily member 19 (TNFRSF19) [118, 119], epidermal growth factor receptor (EGFR) [34], G protein-coupled receptors (GPCRs) [120], and neurokinin-1 receptors (NK1Rs) [121], have been implicated in the induction of paraptosis. GPCR activation by α-hederin in cell paraptosis is mediated by the phospholipase C (PLC)–β3–IP3R–Ca^2+^–protein kinase C alpha (PKCα) pathway in association with increased Ca^2+^ flux to activate MAPK signaling (JNK, p38 MAPK, and ERK) [120]. The synthesized compound 4-PQBH, which specifically binds to Nur77, an orphan nuclear receptor, was shown to induce cytoplasmic vacuolization and cell paraptosis by provoking Nur77-mediated ER stress and autophagy [122]. The expression of TRIP13 (AKT-regulated thyroid receptor-interacting protein 13) is also involved in resistance to the EGFR inhibitor osimertinib-induced paraptosis in glioblastoma cells [34]. The activation of the neurokinin-1 receptor (NK1R) by its ligand, the neuropeptide substance P, also induced cell paraptosis in hippocampal, striatal, and cortical neurons [121] (Fig. 2).

### Transcription and translation upregulation

Paraptosis is completely abolished by the transcription inhibitor actinomycin D and translation inhibitor cycloheximide, suggesting that both transcription and translation are essential for paraptotic progression [1, 8, 9, 26, 35–38]. Treatment with cycloheximide prevents vacuolization and cell death by relieving the accumulation of misfolded proteins and increasing proteotoxicity [36, 95, 123]. In addition to a general response to ER stress, several effectors of the UPR, such as ATF4 and CHOP, serve as transcription factors or cofactors to facilitate selective transcription activity [8, 30, 81]. Additionally, cell cycle arrest has been observed in paraptotic induction [36, 38, 124, 125]. CDK7 and CDK9 are involved in the regulation of RNA polymerase II by interacting with the transcription initiation factor TFIIH and the transcription elongation factor p-TEFb complex to facilitate RNA polymerase II-based transcription [126]. CPYPP, cyclosporin A, and curcumin have been shown to induce CDK7 and CDK9 activation and subsequently RNA polymerase II by increasing the phosphorylation of Rpb1 (the largest subunit of RNA polymerase II) at the Ser2 and Ser5 residues. The activation of the CDK7/CDK9–Rpb1 pathway transcriptionally upregulates the expression of ER stress- and UPR-related genes, including BiP, PERK, IRE1α, GADD34, GADD45α, ATF3, and CHOP. This transcriptional activation also increases the abundance of HSPs, such as HSP27, HSP40, HSP70, and HSP105, which interact with CDK7/CDK9–Rpb1 to form a functional complex in a positive feedback loop to amplify transcriptional activity and exacerbate paraptotic progression [36]. The combination of everolimus and ginsenoside Rh2 has significant anti-lung cancer efficacy both in vitro and in vivo. This anticancer effect is mediated by c-MYC-dependent upregulation of TRIB3 expression, which increases the interaction between TRIB3 and p62 in aggresomes, consequently triggering cell paraptosis [127]. In addition, CREB-binding protein (CBP), Ku70, BAX, and NOX2 reportedly form a transcriptional network that prevents necrosis, paraptosis, and apoptosis in human melanoma. CBP and Ku70 are negative regulators of NOX2. Knocking down CBP or Ku70 provokes cellular ROS, leading to cytoplasmic vacuolization, cell cycle arrest, and eventually paraptosis [128]. During the development of paraptosis, increasing gene and protein synthesis is apparently a compensatory attempt to correct protein misfolding. However, due to impeded thiol-disulfide homeostasis and redox status, excessive de novo protein synthesis results in the accumulation of more unidentified and misfolded proteins in the ER, leading to an overload of proteasome degradation and eventually proteolytic stress in the ER and nuclear stress to provoke overt paraptosis.

### Heat shock proteins (HSPs)

HSPs function as chaperones in various aspects of maintaining cellular proteostasis, including the folding of newly synthesized polypeptides, the folding of misfolded proteins, the assembly of protein complexes, the degradation of misfolded proteins, and the dissociation of protein aggregates [129]. In addition to their housekeeping functions, HSPs also play a role in cellular responses to various stresses. Under normal conditions, heat shock factors (HSFs) are inactively bound to HSPs. Upon stress, HSFs dissociate from HSPs, oligomerize, and translocate to the nucleus to induce the transcription of various HSP genes [130], which are classified by molecular weight into the HSP90, HSP70, HSP40, and small HSP families. They form a network to ensure proteostasis through protein folding, stabilization, complex assembly/disassembly, aggregation/disaggregation, transportation/translocation, and degradation [131].

In the CPYPP-induced paraptosis of MDA-MB-435 melanoma cells and MDA-MB-231 breast cancer cells, several HSPs, including HSP27, HSP40, HSP70, and HSP105, are transcriptionally upregulated [36]. ML346, an HSP79 activator [132], significantly increases the expression of HSP27, HSP40, HSP70, and HSP105 in both the cytoplasmic and nuclear compartments via CDK7–CDK9 activation. ML346 promotes protein ubiquitination and induces cytoplasmic vacuolization and cell paraptosis ER stress sensors and response proteins are also transcriptionally upregulated by ML346. Notably, HSPs were further shown to functionally complex with CDK7/CDK9 to amplify paraptotic signaling [36]. Additionally, HSP60 and HSP70 have been implicated in mediating paraptosis in human U251MG glioma cells overexpressing mM-CSF [133]. Interestingly, the HSP inhibitor VER155008 induces paraptosis in anaplastic thyroid carcinoma cells, a process that requires de novo protein synthesis. VER155008 causes dilation of the ER and increases the mRNA abundance of BiP and CHOP [27]. In macrophage-mediated T9 glioma cell killing, BKCa activation upregulates HSP60, HSP70, HSP90, and gp96, which may act as signals to activate the immune response [93].

### ALG-2-interacting protein (ALIX)

ALIX, also known as AIP1, is a ubiquitously expressed cytoplasmic protein that is located mainly in phagosomes and exosomes. Alix can interact with the Ca^2+^-binding protein ALG-2 (apoptosis-linked gene 2) to induce cell death [134]. Inhibiting ALG-2 expression shields cells from death triggered by various stimuli. The overexpression of the C-terminal half of ALIX/AIP1, which includes the ALG-2-interacting domain, protects cells from apoptosis but induces cytoplasmic vacuolization [135]. However, ALIX is considered an inhibitor of paraptosis by impairing IGF1R-activated MAPK signaling [115]. Several compounds, including α-hederin [120], nitrovin [40], rifampicin [35], morusin [89], kaempferide [136], epimedokoreanin B (EKB) [137], curcumin [41], curcuminoid B63 [30], DHW-221 [124], and YRL1091 [113], have been shown to inhibit paraptosis by repressing ALIX expression. The rescue of cell paraptosis by ALIX is mediated by ROS, as ALIX overexpression attenuates curcuminoid B63-induced paraptosis in cancer cells [30].

### Valosin-containing protein (VCP/97)

VCP is a hexameric AAA + ATPase crucial for various cellular processes, including ERAD, mitochondrial-associated degradation, and the UPS [138]. VCP inhibition by pharmacological compounds or siRNAs induces paraptosis in breast cancer cells by restoring the translation of ATF4 and DDIT4 via eukaryotic translation initiation factor 3 subunit D (eIF3d). This effect is enhanced by hyperactivation of the mammalian target of rapamycin (mTOR)-Akt signaling pathway [139].

### Feline leukemia virus subgroup C receptor 1a (FLVCR1a)

FLVCR1a functions as a heme exporter essential for proper angiogenesis [140]. Heme, a complex of iron and protoporphyrin IX, is required for many biological processes, including oxygen transport, electron transport, detoxification, signal transduction, and iron metabolism 2 [141]. Silencing FLVCR1a in human endothelial cells increases heme levels, causing paraptotic cell death through ROS provocation and ER stress, as evidenced by the significant upregulation of PERK, BiP, and GRP94 expression, thereby impairing angiogenesis [142].

### Autophagy and microtubule-associated protein 1A/1B-light chain 3 (LC3)

The autophagy-lysosome system has been demonstrated to influence paraptosis. 8-p-Hydroxybenzoyl tovarol (TAW), a germacrene-type sesquiterpenoid isolated from *Ferula dissecta* (Ledeb.), was found to induce the autophagic process and antagonize its paraptotic effects on HeLa cells. This effect was enhanced by the mTOR inhibitor rapamycin and suppressed by the autophagy inhibitor 3-methyladenine. Loss of Beclin 1, an autophagic regulator, promotes TAW-induced paraptosis by preventing autophagy [143]. LC3 is a ubiquitous soluble protein in mammalian cells with a critical role in autophagy. During this process, LC3-I is recruited to autophagosomes and conjugated to phosphatidylethanolamine to form LC3-II. Subsequently, autophagosomes fuse with lysosomes to form autolysosomes, where intra-autophagosomal components are degraded by lysosomal hydrolases [18, 144]. SQSTM1/p62, a cargo receptor for selective autophagy, recognizes and binds ubiquitinated proteins and organelles, targeting them for degradation [145].

Paraptosis has been associated with elevated LC3B-II levels [146]. Several agents have been found to be capable of causing cell paraptosis via the induction of MAPK, ROS, ER stress, and cytoplasmic vacuolization, accompanied by upregulated LC3-II expression, including aloperine [125], 5-deoxy-Δ12,14-prostaglandin J2 (5d-PGJ2) [146], dimethoxycurcumin [8], manumycin A [109], YRL1091 [113], 6-shogaol [31], honokiol (HNK) [37], 2’-hydroxyretrochalcone (HRC) [147], and N-(2-hydroxyethyl)formamide compound 4a [148]. However, in the paraptosis process, the role of increased LC3-II and its connection with autophagic flux remains controversial. Aloperine, a natural alkaloid, inhibits late-stage autophagy in glioblastoma cells by targeting lysosomes, leading to increased LC3B-II and p62 protein expression [125]. This aloperine-mediated paraptosis is regulated by the autophagy inhibitor 3-methyladenine, indicating that autophagic flux enhances paraptosis [125]. 5d-PGJ2 upregulates LC3-II expression. LC3-II knockdown attenuates vacuolation and promotes viability in human colon cancer cells (HCT116) and breast cancer cells (MDA-MB-231). However, despite the upregulation of LC3-II, the autophagy inhibitors 3-methyladenine, and phosphatidylinositol 3-kinase (PI3K) inhibitors wortmannin and LY294002 do not affect LC3-II expression, suggesting that autophagic influx is independent of LC3-II levels [146]. Manumycin A is a natural antibiotic derived from *Streptomyces parvulus* [149]. It upregulates LC3-II and p62 during paraptosis. LC3-II deficiency reduces growth and confers protection against manumycin A-induced cytotoxicity and tumor growth in breast cancer cells. However, the absence of an effect with wortmannin and 3-methyladenine indicates that this manumycin A-induced paraptosis is not associated with autophagic flux, and LC3-II accumulation enhances paraptosis. Upregulation of LC3-II is also essential for YRL1091-induced paraptosis in breast cancer cells [113]. Similarly, 6-shogaol, honokiol, and 2’-hydroxy-retrochalcone induce non-autophagic paraptosis, accompanied by increased LC3-II and p62 levels [31, 37, 147]. The combination of everolimus and ginsenoside Rh2 increases c-MYC-mediated accumulation of tribble homolog 3 (TRIB3)/p62^+^ aggresomes, triggering non-autophagic paraptosis [127]. Table 3 provides a summary of the association between LC3-II, p62, and autophagic flux induced by these compounds in the context of paraptosis.

**Table 3:** The association of LC3-II, p62, and autophagy by compounds in paraptosis

Compounds	Cancer types	Expression of autophagy-related proteins	Effect of autophagy inhibitors on paraptosis	Reference
8-p-Hydroxybenzoyl tovarol (TAW)	Cervical cancer cells (HeLa)	Upregulation: Beclin-1, LC3-II Downregulation: p62	Autophagy-dependent TAW-induced autophagy antagonized its paraptosis induction Cell viability inhibition was enhanced by 3-methyladenine but attenuated by rapamycin	[143]
Aloperine	Glioblastoma (U87, A172, and GL261)	Upregulation: LC3B-II, p62	Autophagy-dependent Aloperine induced paraptosis by promoting autophagy Cell viability inhibition and LC3B-II expression were attenuated by 3-methyladenine Aloperine directly targeted lysosomes, weakening their acidic conditions	[125]
5-deoxy-Δ^12, 14^-Prostaglandin J_2_, (5d-PGJ2)	Human colon cancer cells (HCT116) breast cancer cells (MDA-MB-231)	Upregulation: LC3-II	Autophagy-independent, LC3-dependent Autophagy inhibitor (3-methyladenine) and PI3K inhibitor (wortmannin and LY294002) had no effect on LC3-II expression Knockdown of LC3 attenuated the vacuolation and cell viability inhibition	[146]
Manumycin A	Breast cancer cells (MDA-MB-231, HCC1937, BT-20)	Upregulation: LC3-II, p62	Autophagy-independent, LC3-dependent Cell viability inhibition and vacuolation were not modulated by 3-methyladenine, chloroquine, wortmannin, and LY294002 LC3-II knockdown attenuated ER stress, vacuolation, and tumor growth, and reduced cell viability inhibition	[110]
YRL1091	Breast cancer cells (MDA-MB-231 and MCF-7)	Upregulation: LC3B-II, p62	Autophagy-independent, LC3-dependent Cell viability inhibition and vacuolation were not modulated by 3-methyladenine and chloroquine LC3-II knockdown attenuated both vacuolation and cell viability inhibition	[113]
Dimethoxycurcumin (DMC)	Human breast cancer (MDA-MB-435S)	Upregulation: LC3B-II No effect: ATG5, ATG6 (Beclin-1)	Autophagy-independent, LC3-independent Cell viability inhibition and vacuolation were not modulated by 3-methyladenine, chloroquine, or LC3 knockdown	[8]
6-Shogaol	Human breast cancer cell (MDA-MB-231), non-small lung cancer (A549) cells	Upregulation: LC3-II, p62	Autophagy-independent Cell viability inhibition and vacuolation were not modulated by 3-methyladenine and chloroquine	[31]
2'-hydroxy-retrochalcone (HRC)	Breast cancer cells (MDA-MB-231	Upregulation: LC3-II, p62	Autophagy-independent Cell viability inhibition and vacuolation were not modulated by 3-methyladenine and chloroquine	[147]
Honokiol (HNK)	Acute promyelocytic leukemia (NB4 cells)	Upregulation: LC3-II, p62	Autophagy-independent Cell viability inhibition was not modulated by 3-methyladenine, LY294002, and rapamycin	[37]
Morusin	Epithelial ovarian cancer (A2780, SLOV-3, HO-8910)	Upregulation: LC3-II, p62	Autophagy-independent Cell viability was slightly but statistically significantly promoted by 3-methyladenine and chloroquine Morusin-induced vacuolation was slightly enhanced by 3-methyladenine and chloroquine	[89]
Combination of ginsenoside Rh2 (Rh2) and everolimus (Eve)	Human lung cancer cell lines (NCI-H1975, HCC827)	Upregulation: LC3-II p62	Autophagy-independent, p62-dependent Cell viability inhibition was not modulated by Bafilomycin A1 and chloroquine p62 knockdown attenuated the cytoplasmic vacuolation and cell viability Eve-Rh2 upregulates TRIB3, causing p62 aggresome accumulation, which then led to paraptosis	[127]
N-(2-hydroxyethyl) -formamide compound 4a (4a)	Human hepatocarcinoma cell line (SMMC-7721)	Upregulation: LC3-II, p62	Un-determined	[148]

### Interplay among intracellular compartments in paraptosis

**Fig. 3:**
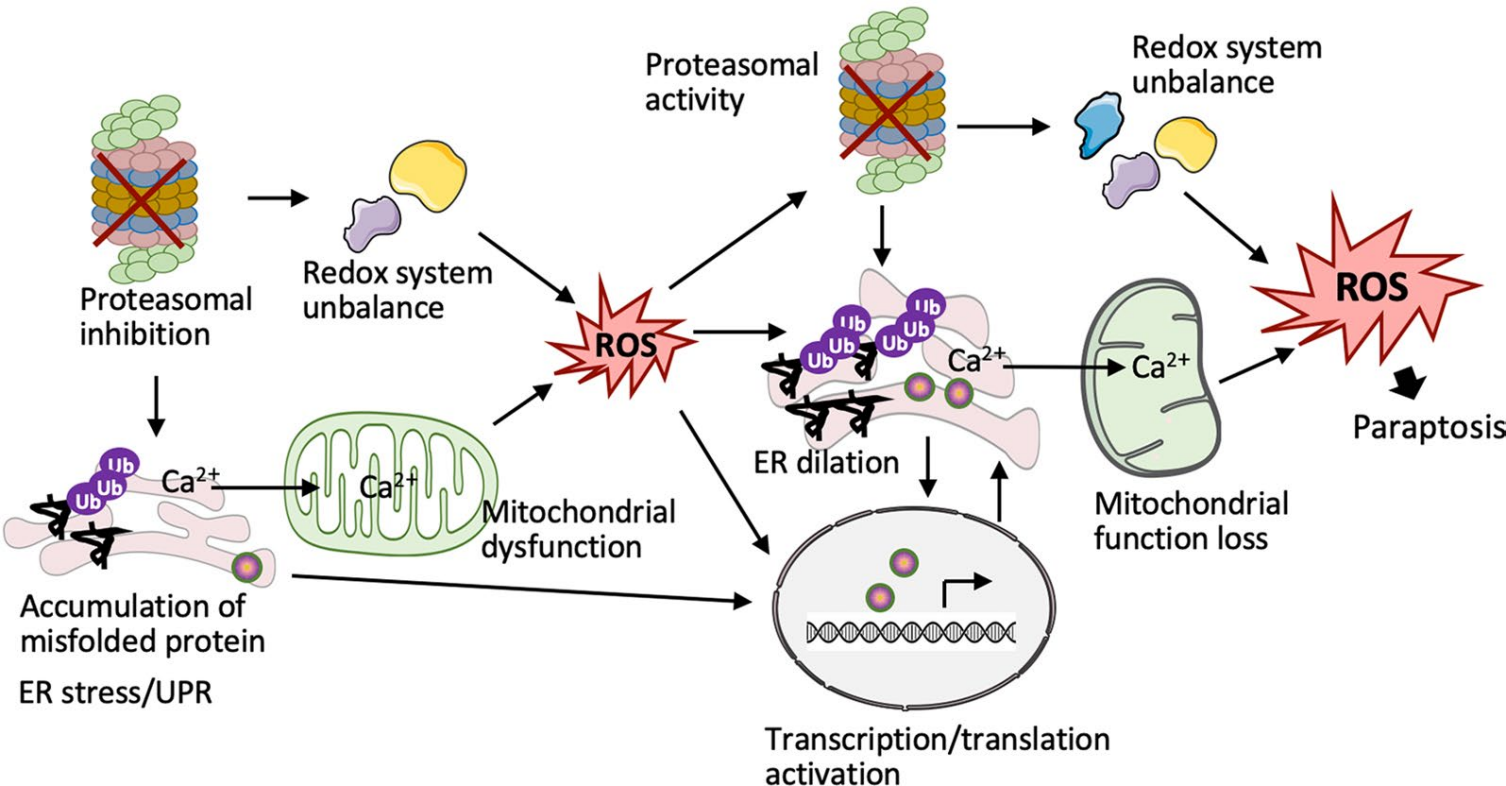
The interplay among intracellular compartments in paraptosis development. Paraptosis can be initiated by proteasomal inhibition, which then leads to endoplasmic reticulum (ER) stress. This stress activates the unfolded protein response (UPR), leading to mitochondrial dysfunction and alterations in nuclear transcription and translation. Mitochondrial dysfunction and imbalances in redox systems increase the production of reactive oxygen species (ROS). Elevated ROS levels further suppress proteasome activity, exacerbate ER stress and the UPR, and activate transcription and translation. This, in turn, further disrupts mitochondrial function and intracellular redox systems, generating more ROS. Additionally, Ca^2+^ in the ER can act as a signaling messenger, communicating between the ER and mitochondria. Furthermore, some ER and UPR proteins act as transcription factors or co-factors, regulating the expression of new genes and proteins and amplifying the paraptotic lethal program

The paraptosis program is coordinated by the proteasome, ER, mitochondria, and nuclear compartments. It is proposed to initiate with the accumulation of misfolded or unfolded proteins, inducing ER stress and subsequently leading to irreversible UPR activation in the ER lumen. This may be attributed to, or exacerbated by, deficient proteasome activation and disruption of ion homeostasis in the ER and mitochondria. These disruptions lead to the interruption of redox homeostasis and mitochondrial dysfunction. Mitochondrial dysfunction and imbalanced redox systems result in ROS overproduction. Meanwhile, some ER stress or UPR proteins act as transcription factors, regulating cellular responses through the synthesis of new genes or proteins. Under pro-oxidant conditions, the overproduced ROS can amplify the lethal effects of paraptosis by inhibiting proteasome activity, increasing ER stress and UPR, and exacerbating mitochondrial dysfunction. This is evidenced by the dilation of the ER and mitochondria and increased transcription/translation activity. The increased ER stress and UPR result from the further accumulation of unfolded/misfolded proteins due to inhibited proteasome activity and promoted transcription/translation, creating a more severe environment and generating more ROS. This ROS then amplifies the paraptotic death signal. In addition to ROS, other signaling molecules, such as Ca^2+^, communicate between cellular compartments, particularly between the ER and mitochondria. UPR proteins can act as transcription factors or cofactors to deliver death signals to the nucleus, where selective transcription and translation are initiated. This process operates as a positive feedback loop that ultimately leads to cell paraptosis (Fig. 3).

## Paraptosis as a therapeutic strategy

### Natural products or small molecules that target paraptosis for cancer treatment

Various natural products and small molecules have been identified as possessing paraptotic activity in cancer cells, both in vitro and in vivo, as reviewed in several articles [2–4, 6, 7]. Most of their paraptotic effects are mediated through proteasome activity/autophagic flux, ER stress/UPR, Ca^2+^/ion balance, and ROS/thiol/redox homeostasis (Fig. 2).

### The combination of paraptosis agents with chemotherapy

Several studies have shown that the combination of chemotherapy or anticancer compounds with paraptosis-inducing agents can enhance therapeutic effects. Examples include afatinib-celastrol against non-small cell lung cancer cells [64], paclitaxel-honokiol against non-small cell lung cancer cells [155], and the mTOR inhibitor jolkinolide B in bladder cancer cells [160]. Non-small cell lung cancer with abundant expression of wild-type EGFR is intrinsically resistant to EGFR tyrosine kinase inhibitors, such as afatinib. The paraptosis inducer celastrol acts synergistically with afatinib to suppress the viability of H23 and H292 lung cancer cells by triggering the paraptotic program. Furthermore, the combination of celastrol and afatinib demonstrated synergistic antitumor activity against H23 xenograft tumors [64]. Notably, H1299 lung cancer cells exhibit intrinsic resistance to paclitaxel. The combination of paclitaxel and honokiol synergistically inhibited H1299 cell viability via paraptotic induction and delayed H1299 xenograft tumor growth [155]. Jolkinolide B enhances the antitumor efficacy of mTOR inhibitors (temsirolimus, rapamycin, or everolimus) against both PTEN-deficient and cisplatin-resistant bladder cancer cells by suppressing AKT signaling and autophagy [160]. Additionally, the combination of the mTOR inhibitor everolimus and the ginsenoside Rh2 was shown to trigger cell paraptosis by promoting c-MYC-mediated TRIB3/p62^+^ aggresome accumulation in lung cancer [127]. DSFoxy, a disulfiram oxy-derivative, cotreated with vitamin B_12b_ activates the paraptotic program to suppress the viability of MCF-7 breast cancer cells [159]. Moreover, chalcomoracin can increase sensitivity to radiotherapy by priming the paraptotic program in human non-small cell lung cancer cells, including H460, A549, and PC-9 cells [32].

**Fig. 4:**
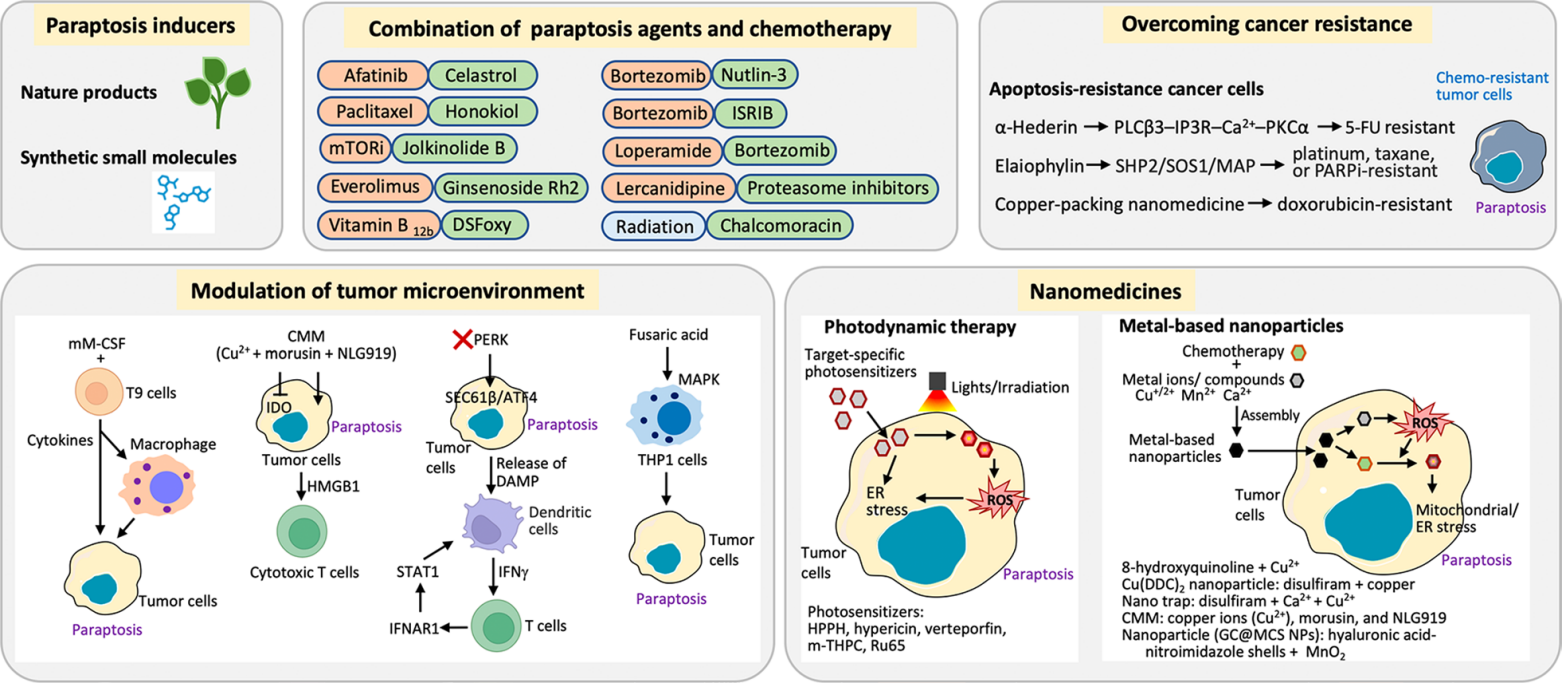
Paraptosis is a promising strategy in cancer treatment, with several demonstrated applications. These include the use of single paraptosis-inducing agents, combining paraptosis agents with clinical chemotherapy and radiation to enhance efficacy, overcoming chemotherapy resistance, modulating the tumor microenvironment, and utilizing nanomedicines. Many natural products and small molecules possess anticancer properties through the induction of paraptosis. Combining these agents with conventional therapies can enhance treatment efficacy by increasing sensitivity to chemotherapy and radiation. Importantly, paraptosis can be induced even in cancer cells that have developed resistance to traditional therapies like chemotherapy. Furthermore, paraptosis induction can activate immune cell activity, including macrophages, dendritic cells, and T cells, through paraptosis-dependent mechanisms. Nanomedicine approaches, such as photodynamic therapy and metal-based particles, utilize photosensitizers and metals, respectively, to increase ROS production, inducing ER stress and mitochondrial dysfunction, which further enhances the effects of chemotherapy. Parts of the figure were created using images with BioRender.com or from Servier Medical Art, which is licensed under a Creative Commons Attribution 4.0 Unported License (https://creativecommons.org/licenses/by/4.0/)

Clinically, inactivation of the proteasome system is an established treatment strategy for patients with multiple myeloma, and several proteasome inhibitors, such as bortezomib, carfilzomib, and ixazomib, have been successfully used to treat hematological malignancies [161, 162]. However, these compounds have shown limited clinical efficacy as monotherapies for solid tumors [163, 164] because innate and acquired mechanisms for resistance development compromise the effectiveness of proteasome-suppressing therapy [165]. Combining proteasome inhibitors with other chemotherapeutic agents can enhance their therapeutic effects on solid tumors. Nutlin-3 is a specific inhibitor of the MDM2‒p53 protein‒protein interaction. The combination of bortezomib and nutlin-3 synergistically promotes cell death, which is accompanied by ER stress, proteasomal inactivation, and cytoplasmic vacuolization in various solid tumors, including breast cancer, colon cancer, cervical cancer, and glioblastoma cells [157]. The integrated stress response (ISR) is a cellular adaptive signaling response activated by various physiological or pathological stimuli in an attempt to maintain homeostasis [166]. The activation of ISR is correlated with resistance to chemotherapy in numerous cancer types [167]. ISRIB, an integrated stress response inhibitor, potentiates bortezomib-induced cytotoxicity by increasing PERK and eIF2α phosphorylation, thereby restoring proteotoxic stress [65]. The anticancer sensitivities of proteasome inhibitors, including bortezomib, carfilzomib, and ixazomib, were enhanced in the presence of lercanidipine, an antihypertensive drug. Lercanidipine potentiates bortezomib-mediated ER stress, ER dilation, and mitochondrial Ca^2+^ overload, causing mitochondrial dilation and paraptosis in cancer cells [156]. Loperamide, which is used to treat diarrhea, overcomes the resistance of colon cancer cells to bortezomib by priming CHOP-mediated paraptosis [152] (Fig. 4).

### Reversing chemotherapy resistance via paraptotic program induction

Intrinsic resistance or acquired resistance to apoptosis significantly limits the efficacy of anticancer treatments [168]. Inducing alternative cell death pathways could offer a promising therapeutic strategy to overcome resistance to apoptosis in cancer cells. α-Hederin, a plant extract from *Akebia trifoliata* (Thunb.), induces paraptosis in 5-fluorouracil-resistant HT-29 colorectal cancer cells via activation of the PLCβ3–IP3R–Ca^2+^–PKCα pathway [120]. Abnormally activated MAPK signaling is commonly found in most cancers and drives the acquisition of drug resistance [169]. Since paraptosis is largely programmed via the ERK and JNK pathways, drug-resistant cancer cells may preferentially respond to paraptotic agents and undergo programmed death. Elaiophylin, a natural C2-symmetric macrodiolide antibiotic, induces cytoplasmic vacuolization and subsequent paraptosis by promoting the SHP2-SOS1 pathway to hyperactivate MAPK signaling [117]. The induction of cell paraptosis by copper-containing nanomedicine represents a promising strategy for addressing tumors resistant to apoptosis [101, 170]. Copper complexes, Cu(thp)4 and Cu(bhpe)2, at micromolar concentrations, can inhibit the growth of a variety of cancer cells, including lung (A549), breast (MCF-7), liver (HepG2), colon (CaCo-2, HCT-15), and cervix (HeLA) lines, as well as cisplatin-resistant variant ovarian adenocarcinoma cell lines and doxorubicin-resistant colon carcinoma cell lines. This occurs through G_2_/M cell cycle arrest and paraptosis, accompanied by the loss of mitochondrial membrane potential and ROS generation [171]. Furthermore, the copper complexes Cu(thp)4, in the submicromolar range, exhibit strong antiproliferative effects in colon cancer cells and overcome cisplatin and oxaliplatin resistance via activation of ER stress [100].

### The association of paraptosis and tumor microenvironment

Paraptosis has been linked to tumor immunity. T9 glioma cells overexpressing mM-CSF fail to proliferate in rats and are killed by activated macrophages with glioma-specific immunity. Tumor cells are programmed to undergo paraptotic death via ROS-mediated BKCa activation, leading to cytoplasmic vacuolization and intracellular ATP depletion, thereby impeding tumor growth [93]. However, this effect was not observed with the injection of X-irradiated apoptotic T9 cells. During this process, the heat shock proteins HSP60, 70, 90, and gp96 are upregulated, and HMGB1 is translocated from the nucleus to the periphery [94], which may serve as danger signals to stimulate the immune system. HSPs have been shown to enhance the immune response [172]. Oxidative stress-mediated death pathways can transform nonimmunogenic tumors into immunogenic phenotypes [173]. During the immunogenic transformation process, damage-associated molecular patterns (DAMPs) are cooperatively presented to facilitate an immunotherapeutic effect, such as the externalization of calreticulin to the cell membrane and the secretion of ATP and HMGB1 [174]. These immunogenic phenotypes can recruit macrophages to the tumor site and drive immunogenic cell death by igniting the paraptotic program in tumor cells. This characteristic is also shared by some paraptotic compounds. CMM, a paraptosis agent composed of copper ions (Cu^2+^), morusin, and NLG919 (an indoleamine 2,3-dioxygenase (IDO) inhibitor), was found to induce immunogenic cell death in tumor cells, characterized by extensive vacuolization of the ER and mitochondria. NLG919 inhibits indoleamine 2,3-dioxygenase and thus can remodel the tumor microenvironment to generate immunogenic phenotypes [175].

Cancer cells experience mild to moderate levels of ER stress to resist environmental insults and maintain homeostatic UPR through transcriptional and translational changes to adapt and survive [74]. Intrinsic activation of PERK was observed in tumor-associated T cells and myeloid-derived suppressor cells [176, 177]. Deletion of PERK in cancer cells or pharmacological inhibition of PERK in melanoma-bearing mice was shown to activate antitumor T cell immunity and attenuate tumor growth. Ablation of PERK in ER-stressed malignant cells triggered SEC61β-mediated paraptosis and promoted immunogenic cell death and systemic antitumor responses. The PERK-null tumor-bearing mice exhibited elevated type I interferon production in dendritic cells, which in turn promoted the expansion of immune-competent monocyte-derived dendritic cells [178]. Moreover, fusaric acid was shown to induce immunotoxicity by inducing the paraptotic program in the human acute monocytic leukemia cell line THP-1 through the activation of the MAPK pathway [179] (Fig. 4). These findings suggest that paraptosis plays a crucial role in shaping the immune landscape within the tumor. It can lead to the release of DAMPs, which travel to the tumor site and stimulate an anti-tumor immune response. Furthermore, paraptosis can alter the balance of immune cells within the tumor microenvironment, creating a more favorable environment for immune-mediated tumor control.

### Nanomedicine targeting paraptosis

In the field of nanomedicine, paraptosis is of particular interest for its potential to target cancer cells resistant to apoptosis, a common mechanism by which many therapies fail to kill cancer cells [180, 181]. Nanomedicine can leverage the paraptosis pathway by designing nanoparticles that can selectively deliver paraptosis-inducing therapeutic agents to tumors [181, 182]. Currently, most nanomedicines that induce paraptosis in tumor cells primarily exert their antitumor effects through ROS-mediated oxidative stress, leading to cytoplasmic vacuolization and organelle swelling [181, 182].

#### Photomedicine

Photodynamic therapy (PDT) is a powerful cancer treatment that uses light to trigger chemical reactions in abnormal or mutated cells, ultimately leading to their destruction [182–185]. Photodynamic damage in the ER has been demonstrated to prime paraptotic programs [186]. PDT requires the use of light-sensitive drugs, also called photosensitizers, which, when exposed to a specific wavelength of light, stimulate ROS generation to kill cancer cells [181, 182](Fig. 4). Photodynamic hypericin and verteporfin (a benzoporphyrin derivative) were shown to induce the morphological features of paraptosis with massive cytoplasmic vacuolization in human ovarian carcinoma cells, OVCAR-5 [186]. The formation of vacuoles was abolished by MAPK inhibitors or translation inhibitors at low doses of verteporfin [187]. Several photodynamic therapies have been developed utilizing ER-targeted photosensitizers to activate paraptosis in different cancer cell lines, including the pyropheophorbide HPPH [187], hypericin [188, 189], verteporfin [190–192], and m-tetra(hydroxyphenyl) chlorin (m-THPC) [193]. Furthermore, PDT directly acts on specific compartments, such as the ER, mitochondria, and nucleus, to induce paraptosis initially, followed by priming of the apoptotic program, leading to a substantial increase in the radiation response in HPV-negative head and neck squamous cell carcinoma [192]. The photosensitizer [Ru(bipy)2-dppz-7-methoxy][PF6]2 (Ru65), which is localized in the nucleus, displays cytotoxicity only upon UV-A irradiation. Intriguingly, low-dose UV-A irradiation of DNA-intercalated Ru65 induced a transient DNA damage response, subsequently leading to prolonged cell cycle arrest at the S and G_2_/M phases. This was accompanied by extensive cytoplasmic vacuolization, the UPR, a loss of cell viability, and, eventually, massive cell death [194].

### Metal-based nanoparticles

Metal-based nanoparticles function similarly to photomedicine, but instead of light, they utilize encapsulated metal ions to stimulate ROS generation, often triggered by anticancer drugs [195] (Fig. 4). These nanocomplexes gradually aggregate and accumulate near the ER and mitochondrial vacuoles, activating paraptosis. The copper complex of 8-hydroxyquinoline and Cu^2+^ effectively inhibits proteasome activity, inducing paraptosis. This complex enhances ROS generation, activates the MAPK pathway, and triggers mitochondrial swelling, cytoplasmic vacuolization, and subsequent cell death, overcoming resistance to apoptosis and inhibiting tumor growth [101]. The copper diethyldithiocarbamate (Cu(DDC)_2_) complex, derived from disulfiram and copper ions, induces cytoplasmic vacuolization and cell death in paclitaxel-resistant prostate cancer cells [196].

A “nanotrap” encapsulating disulfiram and Ca^2+^/Cu^2+^ ions is endocytosed by tumor cells, ultimately reaching the lysosome. The subsequent release of Ca^2+^ and Cu^2+^ ions leads to mitochondrial calcium overload, increased hydrogen peroxide production, mitochondrial dysfunction, and paraptotic cell death. The released Cu^2+^ ions are reduced to Cu^+^ by GSH, which catalyzes the formation of toxic hydroxyl radicals through a Fenton-like reaction, inducing apoptosis [103]. CMN, a noncovalent complex of copper ions, morusin, and the indoleamine 2,3-dioxygenase (IDO) inhibitor NLG919, induces paraptosis through the morusin-mediated ER and mitochondrial vacuolization. Simultaneously, NLG919 remodels the tumor microenvironment, enhancing antitumor immunity by activating cytotoxic T cells [175].

Additionally, paraptosis induction can be enhanced by combining various nanoparticle packages. Gambogic acid triggers vacuolization-associated paraptotic cell death by disrupting thiol proteostasis [50]. A nanoparticle (GC@MCS NP) composed of a hypoxia-responsive hyaluronic acid-nitroimidazole shell, MnO2 oxygen modulators, and a reduction-responsive γ-PFGA core delivers gambogic acid and Chlorin e6 into cancer cells. After endocytosis, gambogic acid is released, and MnO_2_ promotes ROS generation, facilitating gambogic acid-induced paraptosis by depleting GSH [104].

## Conclusions and perspectives

Cancer cells exhibit significant heterogeneity, varying in their sensitivity to drugs, resistance to treatment, and responsiveness to the induction of various cell death modalities. Consequently, diverse strategies are required to effectively trigger alternative cell death pathways in different cancer cell populations. Advances in molecular techniques have facilitated the identification and redefinition of numerous cell death mechanisms, including ferroptosis, cuproptosis, pyroptosis, entosis, methuosis, parthanatos, and NETosis, each characterized by distinct activation cofactors and biomarkers [197, 198]. Paraptosis, a unique form of programmed cell death, is distinguished by intricate interactions among intracellular compartments, encompassing the cytoplasm, ER, mitochondria, and nucleus. This complex process is modulated by a multitude of factors, including ER stress, the UPR, UPS, and autophagic flux. Ionic imbalances, ROS, thiol-disulfide homeostasis, and redox status also play key roles. Disruption of any of these components can initiate a cascading series of events, amplifying signaling and culminating in an irreversible state marked by pronounced ER and mitochondrial dilation, which appears as cytoplasmic vacuolization. Emerging studies increasingly highlight the potential of paraptosis as a potent anticancer therapy.

Paraptosis agents, both alone and in combination with existing chemotherapeutic agents, have shown significant therapeutic effects. Furthermore, paraptosis can address the clinical challenge of chemoresistance. Nanotechnology offers promising avenues for enhancing paraptosis. For example, nanoparticles can be engineered to improve the release of ROS or boost the paraptosis-inducing effects of drugs, leading to improved anticancer outcomes. Cancer immunotherapy is considered the most promising and potent treatment for cancer. Beyond its direct effects, paraptosis also holds promise for enhancing cancer immunotherapy. Studies have shown that inducing paraptosis helps to remodel the tumor microenvironment and promote the recovery or activation of immune function. These findings highlight the potential of paraptosis in cancer treatment applications. Operating through mechanisms distinct from apoptosis and other types of cell death, paraptosis offers a new avenue for therapeutic intervention.

The biggest challenge in paraptosis research is the lack of definitive markers and unique signaling pathways, with identification currently relying heavily on cytoplasmic vacuolization. This ambiguity led to its exclusion from the 2018 classification of cell death modalities [197]. Furthermore, researchers primarily study drug-induced paraptosis, while its role in normal physiological processes or diseases remains largely unknown. While some studies suggest paraptosis can remodel the tumor microenvironment and affect immune cells [175, 178, 179], its precise impact on immunotherapy and cellular immunity remains unclear. In particular, no research has yet been published on the relationship between paraptosis and current clinical cancer immunotherapy drugs, such as immune checkpoint inhibitors, cellular immunotherapies, cancer vaccines, monoclonal antibodies, or oncolytic viruses. Despite these challenges, paraptosis offers a potential breakthrough for cancer treatment. This article aims to raise awareness and encourage further research into paraptosis, with the ultimate goal of harnessing its unique mechanisms to develop effective therapies for cancer and other diseases.

## Data Availability

The datasets used and/or analyzed during the current study are available from the corresponding author upon reasonable request.

## Abbreviations

ALIX: Alg-2-interacting protein
ATF: Activating transcription factor
BiP: Binding immunoglobulin protein
BKCa: Big conductance Ca^2+^-activated K^+^ channels
Ca^2+^: Calcium ion
CDK: Cyclin-dependent kinase
CHOP: C/EBP homologous protein
Cl^−^: Chloride ion
CLIC1: Chloride intracellular channel 1
Cu^+^: Cu^2+^ copper ion
EGFR: Epidermal growth factor receptor
ER: Endoplasmic reticulum
GADD: Growth arrest and DNA damage-inducible protein
GSH: Glutathione
HMGB1: High mobility group box 1
IGF1R: Insulin-like growth factor 1 receptor
IP3R: Inositol 1,4,5-trisphosphate receptor
IRE1α: Inositol-requiring enzyme 1α
JNK: Janus N-terminal kinase
K^+^: Potassium ion
LC3: Microtubule-associated protein 1A/1B-light chain 3
MAPK: Mitogen-activated protein kinase
mM-CSF: Membrane macrophage colony-stimulating factor
PERK: Protein kinase RNA-like endoplasmic reticulum kinase
ROS: Reactive oxygen species
RyR: Ryanodine receptor
TrxR: Thioredoxin reductase
UPR: Unfolded protein response
UPS: Ubiquitin‒proteasome system
VCP: Valosin-containing protein
